# Systemic Therapy of Metastatic Melanoma: On the Road to Cure

**DOI:** 10.3390/cancers13061430

**Published:** 2021-03-20

**Authors:** Julian Steininger, Frank Friedrich Gellrich, Alexander Schulz, Dana Westphal, Stefan Beissert, Friedegund Meier

**Affiliations:** Skin Cancer Center at the University Cancer Center and National Center for Tumor Diseases, Department of Dermatology, Faculty of Medicine and University Hospital Carl Gustav Carus, Technical University Dresden, 01307 Dresden, Germany; julian.steininger@uniklinikum-dresden.de (J.S.); alexander.schulz@uniklinikum-dresden.de (A.S.); dana.westphal@uniklinikum-dresden.de (D.W.); stefan.beissert@uniklinikum-dresden.de (S.B.); friedegund.meier@uniklinikum-dresden.de (F.M.)

**Keywords:** melanoma, systemic therapy, Immune checkpoint inhibitors, BRAF inhibitors, MEK inhibitors

## Abstract

**Simple Summary:**

Malignant melanoma is more dangerous than most other skin cancers due to its ability to spread early and aggressively. Until the development of new therapeutic strategies, the median survival of patients with metastatic melanoma was just a few months. Immunotherapy, the first regimen, leading to significant improvement, blocks immune checkpoints, which normally dampen immune responses, enabling our defense cells to recognize and destroy cancer cells again. Immunotherapy achieves long-term survival in about 50% of metastatic melanoma patients. Besides, targeted therapy has also significantly improved the survival of melanoma patients, blocking cell-signaling proteins, which are altered in about 50% of melanomas and lead to uncontrolled tumor cell growth. In addition to the approved regimens, there are numerous new treatment strategies, ranging from modified viruses to personalized immune cells that attack and destroy tumor cells. This review shall give an insight into both already approved regimens and upcoming developments.

**Abstract:**

This decade has brought significant survival improvement in patients with metastatic melanoma with targeted therapies and immunotherapies. As our understanding of the mechanisms of action of these therapeutics evolves, even more impressive therapeutic success is being achieved through various combination strategies, including combinations of different immunotherapies as well as with other modalities. This review summarizes prospectively and retrospectively generated clinical evidence on modern melanoma therapy, focusing on immunotherapy and targeted therapy with BRAF kinase inhibitors and MEK kinase inhibitors (BRAF/MEK inhibitors), including recent data presented at major conference meetings. The combination of the anti-PD-1 directed monoclonal antibody nivolumab and of the CTLA-4 antagonist ipilimumab achieves unprecedented 5-year overall survival (OS) rates above 50%; however, toxicity is high. For PD-1 monotherapy (nivolumab or pembrolizumab), toxicities are in general well manageable. Today, novel combinations of such immune checkpoint inhibitors (ICIs) are under investigation, for example with cytokines and oncolytic viruses (i.e., pegylated interleukin-2, talimogene laherparepvec). Furthermore, current studies investigate the combined or sequential use of ICIs plus BRAF/MEK inhibitors. Several studies focus particularly on poor prognosis patients, as e.g., on anti-PD-1 refractory melanoma, patients with brain metastases, or uveal melanoma. It is hoped, on the road to cure, that these new approaches further improve long term survival in patients with advanced or metastatic melanoma.

## 1. Introduction

Around 5–6 decades ago, a new ideal of beauty in skin color became more and more apparent: tanned skin reflecting a sporty and healthy lifestyle. Thereby increased UV-exposition is regarded as the most important reason that incidence rates of melanoma keep rising dramatically over the past years. Although being far less prevalent than other skin malignancies in total, melanoma is responsible for around 90% of deaths concerning skin cancer.

Melanoma arises from atypically transformed melanocytes whose fetal precursor cells originate from the neural crest. Like other cell types, melanocytes undergo a stepwise process acquiring features summarized as the hallmarks of cancer [1,2]: 1. Self-sufficiency in growth signals; 2. Insensitivity to growth-inhibitory signals; 3. Evasion of programmed cell death; 4. Limitless replicative potential; 5. Sustained angiogenesis; 6. Tissue invasion and metastasis; 7. Deregulation of cellular energetics; 8. Avoiding immune destruction. These pathogenic changes frequently develop from activation of tumor-promoting oncogenes and/or silencing of tumor-suppressor genes by somatic mutations, genomic rearrangements (deletion, amplification), or dysregulated gene expression through epigenetic mechanisms. Mutations in B-Raf protooncogene, serine/threonine kinase (BRAF), NRAS protooncogene GTPase (NRAS), or KIT protooncogene, receptor tyrosine kinase (KIT) genes, as well as loss of tumor suppressor phosphatase and tensin homolog (PTEN) or cyclin-dependent kinase inhibitor 2A (CDKN2A) occur at early stages of malignant transformation [3]. However, loss of E-Cadherin and upregulation of N-Cadherin are characteristic for later stages [3]. The changes associated with the cellular adhesion molecules E-Cadherin and N-Cadherin are part of the epithelial to mesenchymal transition (EMT) and substantially contribute to progression of melanoma cells by promoting detachment from the basal keratinocytes within the epidermis (invasion) and subsequent intravasation into the blood stream (metastasis). Genetic instability and external factors such as ultraviolet (UV)-radiation may accelerate malignant transformation and usually affect relevant survival pathways. In case of melanoma, the most frequently hyperactivated pathways are the mitogen-activated protein kinase (MAPK) and the phosphatidylinositol 3-kinase (PI3K) pathway [4,5,6,7]. However, there is evidence of other pathways being hijacked as well, including but not limited to hepatocyte growth factor (HGF)- or microphthalmia-associated transcription factor (MITF)-signaling [8,9,10,11], for example. In summary, specific sequential steps are required for melanocytes to transform into malignant melanoma and metastasize to lymph nodes and distant organs like the lung, the liver, or the brain. Consequently, each event may worsen patient prognosis, emphasizing the importance of early diagnosis for improved patient outcome.

Until recently, the diagnosis of distant metastases had an unfavorable prognosis with a median survival of 6–9 months [12,13]. The ground-breaking discovery of immunotherapy and targeted therapy to be suitable defense mechanisms revolutionized the standard of care and led to new optimism in treating melanoma. Besides the already existing regimens, there is a variety of promising approaches for new therapeutic options. Here, we provide an overview to offer an insight into approved treatment options as well as upcoming developments.

## 2. Material and Methods

This review summarizes clinically relevant data from prospective and retrospective trials on the treatment of metastatic melanoma with targeted therapies and immunotherapies, including recent data presented at the ASCO (American Society of Clinical Oncology) and ESMO (European Society for Medical Oncology) Annual Meeting.

## 3. Immune Checkpoint Inhibitors

In 1995, James P. Allison and colleagues revealed the purpose of cytotoxic T-lymphocyte-associated antigen 4 (CTLA-4), an immune checkpoint molecule, which is able to downregulate steps in T cell activation [14,15]. It has been hypothesized that, by blocking these inhibitory immune checkpoint molecules, it is possible to stimulate the immune response against cancer cells.

**Figure 1:**
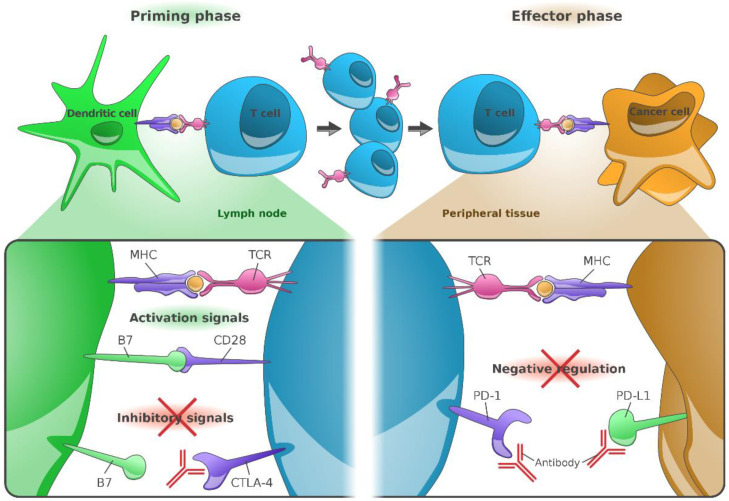
Immunological mode of action of anti-CTLA-4 (CD152), anti-PD-1 (CD279), and anti-PD-L1 (CD274) monoclonal antibodies. The major histocompatibility complex (MHC), present on the surface of cancer cells or dendritic cells, presents peptides derived from tumor-associated antigens (TAAs), which are recognized by T-cells via their T-cell receptors (TCR). Additional cell signaling is provided by the co-stimulatory molecules B7-1 (CD80) or B7-2 (CD86). Both factors are required for T-cell priming. Once activated, T-cells upregulate CTLA-4 expression on their cell surface; in contrast, binding of CTLA-4 to B7 receptors of dendritic cells results into inhibition of T-cell activation. Anti-CTLA-4 directed antibodies hence block inhibitory signaling and restore T-cell activation in lymph nodes. Continued stimulation results into upregulation of PD-1 receptors in T-cells, their (parallel) inhibition prevents the interaction of PD-1 with its ligand, PD-L1. Due to the omission of such negative regulation, tumor cells expressing PD-L1 can again be identified by effector T-cells [22]. Figure adapted from Ribas A [22] and created by Gellrich FF, first published in *J. Clin. Med.* [23].

Ipilimumab, a monoclonal antibody against CTLA-4 (Figure 1), was the first drug to demonstrate a significant improvement in OS of advanced melanoma in a phase 3 study (Table 1) [16]. Over time, however, several trials demonstrated that ipilimumab monotherapy is inferior to antibodies directed against programmed death 1 (PD-1) receptor [17,18,19,20].

Besides blocking the inhibiting immune checkpoint CTLA-4 and thereby bolstering immune response against cancer cells, a second target became apparent in the last decades: In 1992, Tasuku Honjo and colleagues discovered the programmed death 1 (PD-1) receptor [21], expressed on a variety of immune cells, such as T cells, B cells, monocytes, dendritic cells, and tumor-infiltrating lymphocytes (TILs).

PD-1, interacting with its ligand PD-L1 on tumor cells and antigen-presenting cells, and PD-L2 on activated monocytes and dendritic cells, inhibits T cell activity, thereby regulating immune tolerance. Physiologically, the PD-1/PD-L1 pathway is needed as a regulatory checkpoint in order to slow down the degree of inflammation. Various tumors, especially melanomas, overexpress PD-L1 to escape from the inflammatory process and generate an immunosuppressive tumor environment. Blocking either PD-1 or PD-L1 with antibodies facilitates an effective antitumor immune response to kill tumor cells.

The discovery of immune checkpoint blockade led to a new era in fighting cancer. To appreciate this ground-breaking discovery, the Nobel Prize for Physiology or Medicine was awarded jointly to James P. Allison and Tasuku Honjo in 2018.

**Table 1:** Relevant clinical trials demonstrating efficacy of immune checkpoint inhibitors and BRAF kinase inhibitors and MEK kinase inhibitors (BRAF/MEK inhibitors) in unresectable/metastatic melanoma.

Trial (NCT n°)	Agents/dose (mg/kg or mg/m^2^)	Phase	Patients	N	Prim. EP	ORR, %	PFS _Med_, mts (HR [95%CI])	OS _Med_, mts (HR [95%CI])	Ref.
Anti-CTLA-4 directed Immunotherapy									
MDX010-020 (NCT00094653)	Ipi 3 + gp100 vs. Ipi 3 vs. gp100 (3:1:1)	III	Unresectable Stage III/IV, pre-treated, HLA-A*0201–pos.	676	OS	6 vs. 11 vs. 2	2.8 vs. 2.9 vs. 2.8 (0.64 [0.50–0.83])	10.0 vs. 10.1 vs. 6.4 (0.66 [0.51–0.87] ^a^	[16]
Anti-PD-1-directed Immunotherapy (Immune Checkpoint Inhibition mono, or in combination with an anti-CTLA-4 antagonist)									
CM-066 (NCT01721772)	Niv 3 q2w + *Placebo* vs. *Placebo* + Dacarbazine 1000 q3w (1:1)	III	Metastatic, untreated, *BRAF* WT	418	OS	40 vs. 14	5.1 vs. 2.2 (0.43 [0.34–0.56])	37.5 vs. 11.2 (0.46 [0.36–0.59])	[24,25]
CM-067 (NCT01844505)	Niv 1 +Ipi 3 (q3w) x4 → Niv 3; Niv 3 alone q2w, vs. Ipi 3 q3w x4 (1:1:1)	III	Unresectable Stage III/IV, untreated	945	PFS and OS (co-primary)	58 vs. 44 vs. 19	11.5 vs. 6.9 vs. vs. 2.9 (0.42 ^b^ [0.31–0.57]) ^c^; (0.57 ^b^ [0.43–0.76]) ^d^		[26,27]
CM-511 (NCT02714218)	Niv 1 +Ipi 3 (q3w) x4 → Niv 3 vs. Niv 3 +Ipi 1 (q3w) x4 → Niv 3 (1:1)	III	Unresectable Stage III/IV, untreated	360	TRAE rate (grade 3–5)	TRAE: 48 vs. 34	8.9 vs. 9.9 (1.06 [0.79–1.42])	NR vs. NR (1.09 [0.73–1.62])	[28]
CM-003 (NCT00730639)	Niv 0.1 vs. Niv 0.3 vs. Niv 1 vs. Niv 3 vs. Niv 10 (all q2w)	I	1–5 prior systemic therapies (ocular melanoma allowed)	107	Safety, ORR	35 vs. 29 vs. 31 vs. 41 vs. 20	3.6 vs. 1.9 vs. 9.1 vs. 9.7 vs. 3.7	16.2 vs. 12.5 vs. 25.3 vs. 20.3 vs. 11.7	[29]
KN-006 (NCT1866319)	Pem 10 q2w vs. Pem 10 q3w vs. Ipi 3 q3w (1:1:1)	III	Unresectable Stage III/IV, ≤1 prior systemic therapy, Ipi-naïve	834	PFS & OS (co-primary)	34 vs. 33 vs. 12	8.4 vs. 3.4 (0.57 [0.48–0.67]) ^e^	32.7 vs. 15.9 (0.73 [0.61–0.88]) ^e^	[30]
KN-001 (NCT01295827)	Pem 2 q3w/Pem 10 q3w/Pem 10 q2w	IB	Advanced or metastatic, pre- or untreated	655	ORR	41 (52, if untreated)	8.3 (16.9 in the 151 untreated patients)	23.8 (38.6 in the 151 untreated patients)	[31]
Herpes simplex virus-1 derived Oncolytic virus									
OPTiM (NCT00769704)	Tal (10^6^ pfu/mL →10^6^ pfu/mL q3w i.l.) vs. GM-CSF 125 µg/m^2^ (2:1)		Unresectable Stage IIIB/IV M1c, ≥1 sub-/cutaneous lesion 10 mm	436	DRR (16 vs. 2)	DRR: 19% vs. 1%	nr	23.3 vs. 18.9 (0.79 [0.62–1.00])	[32]
Targeted therapy (BRAF mono) 16 vs. 2									
BRIM-3 (NCT01006980)	Vem 960 mg td vs. DTIC (1:1)	III	Metastatic, untreated, *BRAF^V600E^*-mut.	675	PFS & OS (co-primary)	48 vs. 5	5.3 vs. 1.6 (0.26 [0.20–0.33])	NR vs. 7.9 (0.37 [0.26–0.55])	[33]
Combined BRAF+MEK blockade									
COMBI-v (NCT01597908)	Dab 150 bd + Tra 2 od vs. Vem 960 bd (1:1)	III	Metastatic, untreated, *BRAF^V600E/K^*-mut.	704	OS	64 vs. 51	11.4 vs. 7.3 (0.56 [0.46–0.69])	NR vs. 17.2 (0.69 [0.53–0.69])	[34,35]
COMBI-d (NCT01584648)	Dab 150 bd + Tra 2 od vs. Dab 150 bd (1:1)	III	Unresectable Stage IIIC/IV, untreated, *BRAF^V600E/K^*-mut.	423	PFS	69 vs. 53	11.0 vs. 8.8 (0.67 [0.53–0.84])	25.1 vs. 18.7 (0.71 [0.55–0.92])	[35,36]
CoBRIM (NCT01689519)	Cob 60 od d1-21 + Vem 960 bd vs. Vem 960 bd + *Placebo* (1:1)	III	Unresectable Stage IIIB–IV, untreated, *BRAF ^V600^*-mut.	495	PFS	68 vs. 45	9.9 vs. 6.2 (0.51 [0.39–0.68])	22.3 vs. 17.4 (0.70 [0.55–0.90])	[37,38]
COLUMBUS (NCT01909453)	Enc 450 od + Bin 45 mg bd vs. Enc 300 mg od vs. Vem 960 mg bd (1:1:1)	III	Unresectable Stage IIIB–IV, un-treated (or progressed after first-line IT), *BRAF^V600E/K^*-mut.	577	PFS	63 vs.51 vs. 40	14.9 vs. × 9.6 vs. 7.3 (0.54 [0.41–0.71] ^f^	33.6 vs. 23.5 vs. 16.9 (0.61 [0.47–0.79] ^f^	[39,40]
NEMO (NCT01763164)	Bin 45 bd vs. Dacarbazine 1000	III	Unresectable Stage IIIB–IV, un-treated (or progressed after first-line IT), *NRAS^V600E/K^*-mut.	402	PFS	15 vs. 7	2.8 vs. 1.5 (0.62 [0.47–0.80])	11.0 vs. 10.1 (1.00 [0.75–1.33])	[41]
Triplett therapy (ICI + BRAF/MEK-blockade)									
IMSpire150 (NCT02908672)	Ate 840 d1,15 + Vem 720 bd + Cob 60 od d1-21 vs. *Placebo* + Vem 960 bd + Cob 60 od d1-21 (*all*: q4w)	III	Untreated, BRAF-mut.	514	PFS	66 vs. 65	15.1 vs. 10.6 (0.78 [0.63–0.97])	not yet reached/reported	[42]
COMBI-I (NCT02967692)	Spa 400 mg + Dab 150 bd + Tra 2 od vs. *Placebo* + Dab 150 + Tra 2 (q4w)	III	Unresectable Stage IIIB–IV, untreated, *BRAF ^V600^*-mut.	532	PFS		16.2 vs. 12.0 (0.82 [0.66–1.03])	NR vs. NR (0.79 [0.59–1.05])	[43]

^a^ i.e., for the comparison of Ipi vs. gp100. ^b^ 99.5%CI. ^c^ i.e., for comparison of Niv + Ipi vs. Ipi. ^d^ i.e., for comparison of Niv + Ipi vs. Ipi. ^e^ i.e., for comparison of Pem (q2w + q3w arms) vs. Ipi. ^f^ i.e., for comparison of Enc + Bin vs. Vem. Abbreviations: →, then (drug) administered until disease progression or unacceptable toxicity; Ate, atezolizumab; bd, twice daily; Bin, binimetinib; Cob, cobimetinib; Dab, dabrafenib; Enc, encorafenib; EP, endpoint; i.l., intralesional; Ipi, ipilimumab; IT, immunotherapy; Niv, nivolumab; NR, not reached; nr, not reported; od, once daily; ORR, overall response rate; OS, median overall survival; Pem, pembrolizumab; PFS, median progression-free survival; q2w/q3w/q4w, all two/three/four weeks; Spa; spartalizumab; Tal, talimogene laherparepvec; Tra, trametinib; TRAE, treatment-related adverse events; Vem, vemurafenib; vs., versus.

### 3.1. Anti-PD-1 Directed Monoclonal Antibodies

In 2014, the U.S. Food and Drug Administration (FDA) approved pembrolizumab and nivolumab as the first anti-PD-1 (CD279) directed monoclonal antibodies (Figure 1) for use in advanced cancers, namely in advanced or metastatic melanoma. Both drugs received EU approval in 2015.

Approval of nivolumab was based on results of the CheckMate 066 phase *3* study (Table 1) [24]. A total of 418 previously untreated patients with advanced melanoma were randomly assigned in two groups either receiving nivolumab or dacarbazine. The objective response rate (ORR) was 40% for nivolumab versus 14% for dacarbazine. In 2019, the 3-year outcomes of the CheckMate 066 study were reported [25]: the 3-year OS rate was 51% for nivolumab versus 22% for dacarbazine, with a median OS of 37.5 months for nivolumab and 11.2 months for dacarbazine, respectively. In the nivolumab group, grade 3 or 4 treatment-related adverse events (TRAEs), as determined by the U.S. National Cancer Institute’s Common Terminology Criteria for Adverse Events (NCI-CTCAE), occurred in 15% compared to 18% in the dacarbazine group.

Three months prior to nivolumab, pembrolizumab had been approved by the U.S. FDA for the treatment of metastatic melanoma. The accelerated approval was based on results of an activity-estimating cohort conducted within the phase 1b KEYNOTE-001 trial [31]. Of the 411 who enrolled in the KEYNOTE-001 trial, 173 had disease progression after previous therapy with ipilimumab or a BRAF inhibitor. These 173 patients randomly received either 2 mg/kg (*n* = 89) or 10 mg/kg (*n* = 84) of pembrolizumab every three weeks until disease progression or until unacceptable toxicity. The ORR was 24% for 2 mg/kg pembrolizumab and 26% for 10 mg/kg pembrolizumab. In 2019, the 5-year follow-up analysis of KEYNOTE-001 was published with data of 655 patients in total (previously treated or treatment-naïve) [44]. The estimated 5-year OS rate was 34% in all patients, with a median OS of 23.8 months. Grade 3 or 4 TRAEs were reported in 17% of patients.

In the phase 3 KEYNOTE-006 trial, pembrolizumab as a single agent in melanoma patients previously untreated for their advanced or metastatic disease was investigated compared to anti-CTLA-4 ipilimumab therapy [45]. The three-arm trial randomized 834 patients either to pembrolizumab 10 mg/kg every 2 weeks or every 3 weeks, or to ipilimumab 3 mg/kg every 3 weeks. The ORR was 34% for pembrolizumab every 2 weeks, 33% for pembrolizumab every 3 weeks and 12% for ipilimumab. The other initially reported trial results are displayed in Table 1. In 2019, a 5-year follow-up analysis was published [30]. The 5-year OS rate was 39% in the combined pembrolizumab group and 31% in the ipilimumab group. Grade 3 or 4 TRAEs occurred in 17% of the patients in the pembrolizumab group and in 20% of the patients in the ipilimumab group.

The efficacy of pembrolizumab and nivolumab has never been directly compared in patients with metastatic melanoma. A retrospective study compared the OS of 888 patients with metastatic melanoma treated with first-line pembrolizumab or nivolumab [46]. No statistical difference in OS was seen between patients treated with pembrolizumab compared with nivolumab. A similar retrospective analysis was done for patients with recurrent or advanced non-small cell lung cancer [47]. No significant difference in progression-free survival (PFS) was observed between patients treated with pembrolizumab compared to nivolumab.

#### Summary

According to the current ASCO, ESMO, and NCCN guidelines, patients with advanced melanoma with BRAF-wildtype should either receive a dual therapy with ipilimumab plus nivolumab (see below) or a monotherapy with nivolumab or pembrolizumab. The same recommendation is valid for BRAF-mutant advanced melanoma including an additional option with BRAF/MEK inhibitors [17,18,19,20]. 

### 3.2. Combination of Anti-PD-1 with Anti-CTLA-4 Monoclonal Antibodies

The combined blockade of the targets CTLA-4 and PD-1 is supposed to synergistically stimulate the immune response against cancer cells.

The approval of the dual therapy was based on data of the phase 3 CheckMate 067 trial [26]. A total of 945 therapy-naïve patients with unresectable stage III or stage IV melanoma were randomized in three groups, either receiving ipilimumab plus nivolumab or nivolumab or ipilimumab. The ORR was 58% in the ipilimumab plus nivolumab group, 44% in the nivolumab group and 19% in the ipilimumab group. Moreover, the trial convincingly met its co-primary study endpoints, PFS and OS (Table 1). Important to note is that CheckMate 067 had been powered for a comparison between the combined therapy arm versus ipilimumab, but not versus nivolumab. The long-term follow-up data today serve as a relevant benchmark for survival in advanced melanoma, showing a 5-year OS of 52% for the combined therapy versus 44% for nivolumab monotherapy versus 26% for ipilimumab monotherapy [27]. Rates of TRAEs reported in the 60 months follow-up analysis were similar to results reported before [48]. Grade 3 or 4 TRAEs were observed in 59%, 23%, and 28% of the patients in the dual therapy group, nivolumab monotherapy group, and ipilimumab monotherapy group, respectively. Regarding grade 3 TRAEs, the most common ones were diarrhea both in the ipilimumab plus nivolumab group (9%) and in the nivolumab group (3%) and colitis in the ipilimumab group (7%). Increased lipase was the most common grade 4 TRAE in all three groups. Discontinuation of treatment due to TRAEs was reported in 40% of patients in the dual therapy group, in 13% of patients in the nivolumab group, and in 15% of patients in the ipilimumab group.

A trial important to better elucidate the contribution of each combination therapy compound to toxicity is the CheckMate 511 trial, conducted by Lebbé et al. [28]. CheckMate 511 compared ‘standard’ regimen doses of 3 mg/kg ipilimumab and 1 mg/kg nivolumab as used e.g., in CheckMate 067 with a 1 mg/kg ipilimumab and 3 mg/kg nivolumab regimen. The results showed decreased toxicity but similar efficacy for the reduced ipilimumab and increased nivolumab regimen. It must be mentioned that the study was only powered regarding the comparison of toxicity, but not efficacy. The outcomes of this trial hence backed the assumption that the immunotoxicity of the combination can be reasonably moderated by a diminished ipilimumab dosage.

#### 3.2.1. Dosage Regimen of the Combined Therapy

The present standard of care is four doses of the combined therapy, followed by nivolumab as maintenance therapy. Retrospective data, however, indicated similar results when given fewer doses of the dual therapy due to toxicity. Moreover, it appears that the efficacy and tolerability of ipilimumab and nivolumab mostly depend on the first two doses. At ASCO 2020, a phase 2 trial with a total of 60 patients with metastatic melanoma appeared to support this hypothesis [49]: participants initially received two doses of the dual immunotherapy. Patients with a complete response (CR), a partial response (PR), or with stable disease (SD), if confirmed by a CT scan after 6 weeks, instantly switched to maintenance therapy with nivolumab monotherapy. Patients with progressive disease (PD) completed full regimen with 4 doses and afterward initiated maintenance therapy. At week 6, 68% of the 60 patients showed no progression. Among the 19 patients (32%) with progression after 6 weeks, none of the patients had subsequently a RECIST response in week 12. In total, 77% of the patients received only one or two doses of the combined therapy.

The best overall response (BOR) at week 12 was 48%, with CR in 5% and PR in 43%. Grade 3 or 4 TRAEs occurred in 57%. Immunologic effects were measured via Ki67 and ICOS in CD8+ cells in peripheral blood: Ki67, a cell proliferation marker, and ICOS, a T cell costimulatory receptor mainly expressed on activated cytotoxic T cells, memory T cells and regulatory T cells, increased after the first dose of the dual therapy. Afterward, levels were not further rising. These findings implicate that immunologic effects start after the first dose of the dual therapy and do not increase afterward.

#### 3.2.2. Summary

In summary, the dual therapy of ipilimumab and nivolumab appears to be superior either to anti-CTLA-4 monotherapy or to anti-PD-1 monotherapy. The current ASCO, NCCN, and ESMO guidelines recommend ipilimumab plus nivolumab as a reasonable treatment in patients accepting the significantly increased toxicity rates in the combination [17,18,19].

### 3.3. Resistance

Comparing the ORR of the different immunotherapy trials in melanoma, it must be noticed that approximately 40–60% of patients do not achieve significant therapeutic responses under anti-PD-1 monoclonal antibodies [25,27,30]. Moreover, 20% of patients with a CR and 60% of patients with a PR appear to experience disease progression within 5 years after therapy due to acquired resistance. A retrospective multi-center study investigated the outcome of 330 patients resistant to anti-PD-1 antibodies (70% primary and 30% acquired resistance) and therefore receiving ipilimumab plus nivolumab or ipilimumab monotherapy [50]. The ORR were 31% and 12%, respectively. After one year, the PFS and OS were 27% and 57% in the combined group and 13% and 38% in the monotherapy group.

A phase 2 trial, investigated a combined therapy with ipilimumab 1 mg/kg and pembrolizumab 200 mg in 70 patients with progression after anti-PD-1 therapy [51]. The response rate (RR) was 31% and median PFS was 4.7 months. Median OS was 24.7 months.

In conclusion, dual immunotherapy appears to show antitumor activity even after *acquired anti-PD-1 resistance.*

### 3.4. Duration of Therapy

Today, an open question for debate remains the optimal duration of ICI therapy.

Data of the KEYNOTE-006 trial suggest, that there is no need to treat patients continuously: a post-hoc 5-year analysis published in 2019 demonstrated that the majority of patients (74%) completing protocol-specified treatment of 2 years of pembrolizumab with at least SD as best response to therapy (i.e., 20% with a CR, 67% with a PR, and 13% with SD) had ongoing disease control [30]. At the ASCO meeting 2020, an update analyzing outcomes of long-term responders in KEYNOTE-006 was presented [52]: the 3-year OS–as accounted for from pembrolizumab therapy completion date (i.e., after 2 years of therapy)—was 100% for patients with a CR, 95% for patients with a PR, and 67% for patients with SD.

A real-world retrospective cohort study investigated the outcome of 185 patients with metastatic melanoma who electively stopped anti-PD-1 therapy with pembrolizumab or nivolumab in the absence of progressive disease or toxicity [53]. At the time of treatment discontinuation, the BOR was CR in 63%, PR in 24%, and SD in 9% of patients. After stopping treatment and a median follow-up of 18 months, 78% of patients were progression-free. The risk of tumor progression following treatment discontinuation was significantly lower in patients with a CR (14%) compared with patients with a PR (32%) or a SD (50%). Patients with a CR, who were treated for less than 6 months, were found to be at significantly increased risk of tumor progression after discontinuing therapy. Further, 32% of patients who were re-treated with pembrolizumab or nivolumab achieved an antitumor response. Anti-PD-1 re-treatment induced a CR in 11% of patients, a PR in 21% of patients, and a SD in 26% of patients.

In a retrospective study, response and duration of response (DOR) of 104 patients with metastatic melanoma who received anti-PD-1 therapy, and baseline and 1-year positron emission tomography (PET) as well as computed tomography (CT) imaging were evaluated [54]. At one year, a minority of patients (28%) showed a CR on CT, while 75% of patients had a complete metabolic response (CMR) on PET. Most patients (68%) with PR on CT showed a CMR on PET. Almost all patients showing a CMR on PET at one year experienced an ongoing therapy response thereafter.

The ESMO consensus conference [20] developed recommendations on immunotherapy duration. In patients achieving a CR, and being treated for >6 months, discontinuation of anti-PD-1 therapy can be considered in case of a confirmed CR at least four weeks later. Patients with a PR should be considered for discontinuation of anti-PD-1 therapy after two years. In the case of SD, stopping anti-PD-1 treatment can be considered after two years. Discontinuation of anti-PD-1 therapy before 2 years can be considered in patients with confirmed radiological PR or SD in case of a CMR on PET after at least 6 months of anti-PD-1 therapy.

Although these recently published data indicate curation-like long term clinical benefit after 2 years of anti-PD-1-directed therapy, comprehensive or even randomized data regarding the optimal length of ICI therapy are still lacking.

### 3.5. Immune-Related Adverse Events

The immunomodulating effects of ICIs generate a specific inflammatory side effect profile, entitled and categorized as immune-related adverse events (irAEs) which may involve all organ systems but affect most commonly the skin, the gastrointestinal plus hepatic tract, and the endocrine glands. Less frequently involved organ systems include the lungs, the heart, and the central nervous, hematologic and musculoskeletal organ systems. Grade 3 or 4 TRAEs are reported in 10–20% of patients receiving nivolumab or pembrolizumab monotherapy, but in 50% and more of patients treated by combined immune checkpoint blockade with ipilimumab and nivolumab [25,27,30,44]. The ASCO, the ESMO, and the NCCN have developed guidelines for the management of irAEs [55,56,57]. 

Very importantly, clinicians should monitor patients closely when ICIs are given, to ensure early detection and competent management, as irAEs may lead to loss of organ function or even death.

## 4. T-VEC

**Figure 2:**
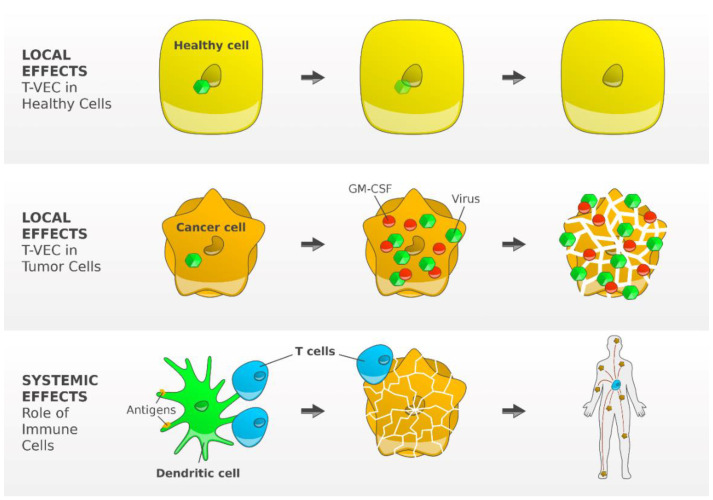
Mode of action of T-VEC, a genetically modified type 1 herpes simplex virus. The functional deletion of one of the two excised, non-essential viral genes, the gene herpesvirus neurovirulence factor (ICP34.5) is attenuating viral pathogenicity and thereby enhancing tumor-selective replication. Inside a normal cell, the virus cannot replicate but may replicate in tumor cells, inducing granulocyte-macrophage colony-stimulating factor (GM-CSF). Subsequent tumor cell lysis results into release of the virus, GM-CSF, and TAAs, which trigger the activity of dendritic cells resulting finally in an efficient induction and activation of tumor-reactive T cells [58]. Figure adapted from Andtbacka R [58] and created by Gellrich FF, first published in *J. Clin. Med.* [23].

Talimogene laherparepvec (T-VEC), a genetically modified herpes simplex virus of type 1 (Figure 2), is the first oncolytic virus therapy approved by the U.S. FDA and the EMA for use as a local treatment of unresectable melanoma of stage IIIB, IIIC, or IVM1a.

Due to deletion of the infected cell protein (ICP) 34.5 gene, which enables neurovirulence, the virus has decreased pathogenicity with promoted selective replication in tumor cells [59]. Immunogenicity is supported by several factors. Deletion of the viral ICP47 gene gives antigenic viral and tumor-associated peptides access to MHC class I complexes, leading to reinforced host immune responses. Insertion of two copies of the human GM-CSF gene attracts local dendritic cells [60]. After injection, T-VEC is expected to replicate within the infected tumor cell. The subsequent lysis releases various fragments, e.g., tumor-associated antigens and GM-CSF, which initiate a local immune response and enhance dendritic cell migration. The dendritic cells trigger a systemic T cell response by presenting antigens to specific CD4+ cells and CD8+ cells in the regional lymph node [61].

The OPTiM trial enrolled 436 patients with injectable but not surgically resectable melanoma metastases either receiving intralesional T-VEC or subcutaneous GM-CSF (Table 1) [32,58,62,63]. The durable response rate, primary endpoint of the trial, was significantly higher in patients treated with T-VEC (19% vs. 1%). The most common TRAEs observed with T-VEC were influenza-like illness, pyrexia, chills, fatigue, and nausea. Grade 3 or 4 TRAEs were reported in 11% of patients treated with T-VEC and in 5% of patients treated with GM-CSF.

Data of 21 patients in phase 1b of the MASTERKEY-265/KEYNOTE-034 study, investigating the combination of T-VEC with pembrolizumab in patients with metastatic melanoma, were presented at ASCO 2016 [64]. After a median follow-up of 33 weeks, the confirmed ORR was 48%, and the unconfirmed ORR was 57%. Grade 3 or 4 TRAEs occurred in 33% of patients. Recruitment of the phase 3 of the MASTERKEY-265/KEYNOTE-034 study has been terminated and the primary completion date is estimated in 2022.

### Summary

So far, the current ASCO and ESMO guidelines do not classify T-VEC as an equivalent therapeutic option for advanced melanoma. Yet, for patients with injectable, unresectable metastases, who do not desire the recommended systemic therapies, T-VEC may act as a primary therapeutic alternative [17,18]. Several studies with T-VEC are ongoing.

## 5. Targeted Therapy

**Figure 3:**
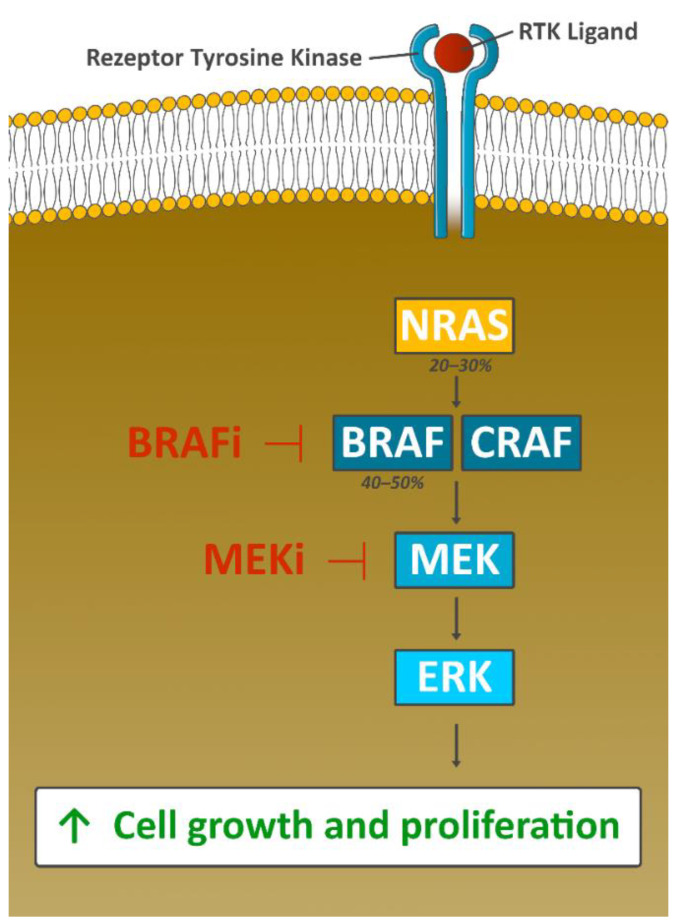
Oncogenic function of the MAPK signaling pathway. Genetic aberrations in this pathway are found in a vast majority of melanomas, with driver mutations in BRAF (V600E or V600K) occurring most often. The activation of BRAF results into phosphorylation of MEK and the activation of downstream MAP kinases like ERK. The pathway co-regulates tumor cell proliferation and cell survival; inhibition of BRAF downregulates the oncogenic function of MAPK signaling [33]. However, resistance to BRAF therapy is observed in about 50% of *BRAF*-mutated patients within 6–7 months after start of therapy. The CRAF-mediated reactivation of MAPK signaling pathway can be effectively blocked by the additional use of a MEK inhibitor (combined BRAF-MEK blockade), prolonging time to resistance development considerably. Consequently, anti-BRAF directed monotherapy is no longer used today. Figure adapted from Jenkins RW [67] and created byGellrich FF.

The discovery of mutations of the BRAF gene in human cancer cells in 2002 laid the groundwork for the targeted therapy of melanoma [65]. Activating mutations in BRAF are found in 40–50% of melanomas (Figure 3). In 2011, a randomized phase 3 trial showed the superiority of the BRAF inhibitor vemurafenib over dacarbazine in OS, PFS, and ORR, and was approved for the treatment of unresectable or metastatic BRAFV600E-mutated melanoma (Table 1) [33]. Although monotherapy with the BRAF inhibitor vemurafenib improves OS, responses are often short-lived. Following the discovery of the clinical activity of single-agent MEK inhibition [66], the use of BRAF/MEK inhibitor combinations was evaluated in clinical studies.

### 5.1. Dabrafenib + Trametinib

The combination of the BRAF inhibitor dabrafenib and the MEK inhibitor trametinib was the first BRAF/MEK inhibitor combination to be approved for the treatment of advanced melanoma in the USA in 2014, and in the EU in 2015. The approval is based on the COMBI-v trial [34] comparing dabrafenib plus trametinib to vemurafenib and the COMBI-d trial [36] comparing dabrafenib plus trametinib to dabrafenib monotherapy. In the phase 3 COMBI-d trial (Table 1), 423 patients were randomized to receive either dabrafenib plus trametinib or dabrafenib alone. The ORR was 69% for the combination and 53% for dabrafenib alone. TRAEs occurred with similar frequency in both arms and most commonly included pyrexia (52%) in the combination arm and hyperkeratosis (33%) in the dabrafenib-only arm. The number of secondary cutaneous cancers decreased with the combination therapy [36]. In the phase 3 COMBI-v trial, 704 patients received either a combination of dabrafenib and trametinib or vemurafenib as first-line therapy (Table 1). The ORR was 64% for the combination and 51% for vemurafenib alone. Rates of severe adverse events (AEs) and study drug discontinuation were similar in both arms [34]. The 5-year OS rate for both, pooled trials together was 34% for the combination arm. The 5-year OS rate was higher among the patients who had a normal lactate dehydrogenase (LDH) at baseline (43%) than among those with an elevated level (16%). In patients with normal LDH and distant metastases in <3 organ sites, the estimated 5-year OS rate was 55%. Further, 19% of the patients who had the BRAF/MEK inhibition achieved a CR—for these patients, the OS rate was 71% at 5-years [35].

### 5.2. Cobimetinib + Vemurafenib

Cobimetinib is another MEK inhibitor developed for the treatment of advanced melanoma in combination with the BRAF inhibitor vemurafenib. In the phase 3 pivotal coBRIM study, 495 patients with previously untreated advanced melanoma were randomized to receive either vemurafenib plus cobimetinib, or vemurafenib alone [37]. ORR was 68% for vemurafenib plus cobimetinib and 45% for vemurafenib alone. Median OS was 22.3 months for the combination versus 17.4 months for vemurafenib (Table 1) [38]. Extended follow-up demonstrated a 4-year OS rate of 35% in the combination group versus 29% in the control group [68]. Grade 3 or 4 TRAEs were slightly more frequent in the combination group (65% vs. 59%). The toxicity profile of vemurafenib plus cobimetinib differs from that of dabrafenib plus trametinib. Diarrhea, nausea, fatigue, rash, liver enzyme abnormalities, and photosensitivity (caused by vemurafenib) are more likely to occur with vemurafenib plus cobimetinib, while pyrexia is more likely to develop with dabrafenib plus trametinib [67].

### 5.3. Encorafenib + Binimetinib

A third combination of a BRAF plus MEK inhibitor, encorafenib and binimetinib, is authorized today. The pivotal, three-arm phase 3 COLUMBUS trial randomized 577 patients either to encorafenib plus binimetinib, to encorafenib, or—as verum-testing control group—to vemurafenib only [39]. Whilst initially only data for PFS as co-primary endpoint were published, OS values for the three study groups were reported (Table 1) after a prolonged follow-up period [40]. The results were best for the combination group with an ORR of 63% vs. 51% vs. 40% and a median OS of 33.6 months vs. 23.5 months vs. 16.9 months. Increased γ-glutamyltransferase (9%), increased creatine phosphokinase (7%), and hypertension (6%) were the most common grade 3 or 4 TRAEs reported for the combination [39]. Since 2018, encorafenib plus binimetinib is approved for use in unresectable or metastatic melanoma with a BRAFV600E or BRAFV600K mutation.

### 5.4. Comparative-Effectiveness of BRAF/MEK Inhibitors

The three treatment regimens approved today for routine clinical use seem to be comparable in terms of efficacy, with ORRs ranging from 60 to 70% and 18-month PFS rates ranging from 30 to 40% [67] with different toxicity profiles. A direct comparison between the studies has not been performed. However, indirect side-by-side analysis of data showed comparable PFS and OS data with a median OS of 22.3 months for vemurafenib plus cobimetinib, 25.6 months for dabrafenib plus trametinib, and 33.6 months for encorafenib plus binimetinib [69]. The approval of three BRAF/MEK inhibitor combinations with different toxicity profiles offers multiple treatment options for patients with BRAF-mutant metastatic melanoma.

### 5.5. Duration of Therapy

The optimal duration of therapy with BRAF/MEK inhibitors is an issue under debate. Targeted therapy is currently often performed for life in the hope of preventing tumor recurrence.

Data on terminating BRAF/MEK inhibitors in patients achieving CR, PR, or SD are limited. In a case series of 12 patients with BRAFV600-mutant metastatic melanoma who achieved CR with BRAF/MEK inhibitors and subsequently discontinued treatment, 50% of patients remained relapse-free whereas 50% of patients recurred at a median of 6.6 months after stopping therapy [70]. In a previous retrospective study, the disease course of 12 patients with metastatic melanoma who stopped BRAF inhibitor therapy after achieving a CR was analyzed [71]. BRAF inhibitor therapy was discontinued due to side effects in seven patients and patient demand in five patients. Six patients were in CR after a median of 17 months after stopping BRAF inhibitor therapy, while the other 6 patients relapsed after a median of 3 months following treatment discontinuation. The 6 relapsing patients were re-treated with a BRAF inhibitor. Three patients achieved a CR, one a SD and one experienced a PD; one patient could not be assessed. The recurrence rate after treatment discontinuation appears to be higher than what is observed with anti-PD-1 therapy. One explanation may be that micrometastases are under cytostatic control during BRAF inhibitor treatment and continue proliferating after treatment discontinuation [72]. In patients who did not relapse, immunological effects of BRAF inhibitors may play a role [73].

In a recent retrospective analysis, 15 patients were treated with a BRAF inhibitor monotherapy and 9 patients with a BRAF/MEK inhibitor combination. The median treatment duration was 59 months. After treatment discontinuation, the median time to PD was 9 months. The risk of PD after treatment discontinuation was 31% and 45% after 12 and 24 months, respectively. In patients who had interrupted treatment with residual disease, a non-significant trend towards a higher risk of relapse was observed compared to patients who had interrupted treatment after reaching CR. In summary, about one third of the patients developed PD within one year of therapy discontinuation after a median therapy duration of 5 years. Therefore, discontinuation of BRAF/MEK inhibitor therapy cannot be recommended at this time. Further biomarker studies may be able to identify patients who are eligible for treatment discontinuation due to persistent toxicity, in particular after achieving CR [74].

### 5.6. Summary

BRAF inhibitors combined with MEK inhibitors are superior to BRAF inhibitor monotherapy in terms of PFS and OS, while toxicity is similar and controllable. The efficacy of the three approved BRAF/MEK inhibitor combinations is comparable [67]. However, their toxicity profiles differ—and may direct clinician’s choice. Discontinuation of BRAF/MEK inhibitor therapy in patients with CR, PR, or SD is not recommended, due to the high relapse rate.

## 6. Sequencing and Combinations of BRAF/MEK Inhibitors and Immune Checkpoint Inhibitors

### 6.1. Mode of Action

Combined BRAF/MEK blockade results in rapid and robust disease control rates and remarkable ORR in BRAFV6oo-mutated metastatic melanoma. However, the DOR is limited in most patients, due to multiple mechanisms of acquired resistance [75]. In contrast, ICIs act more slowly, and sometimes responses are seen after a period of transitional progression (‘pseudo-progression’ due to the massive infiltration of T-cells into the tumor). ICIs induce less frequent but durable responses in approximately 50% of patients [76]. Intriguingly, besides their effect on the MAPK cell signaling pathway, BRAF/MEK inhibitors are thought to also address targets in the cancer-immunity cycle [73,77]. They (1) trigger the release of tumor cell antigens, (2) create an immune stimulatory microenvironment by increasing expression of immune stimulatory molecules and by reducing immunosuppressive cells and cytokines, (3) decrease VEGF expression and enhance infiltration of T cells into the tumor, (4) promote recognition of tumor cells by the immune system through increasing melanoma antigen and HLA I and/or HLA II expression, and (5) intensify reactivity and cytotoxicity of T cells [73]. Results from experimental medicine support the approach to sequence or combine targeted therapy and immunotherapy to trigger synergistic tumor control resulting in rapid, deep, and lasting responses.

### 6.2. First-Line BRAF/MEK Inhibitors versus Immune Checkpoint Inhibitors

Little is known about the best treatment sequence of BRAF/MEK inhibitors and ICIs.

A recent exploratory analysis of survival data from clinical trials with BRAF/MEK inhibitors and ICIs in patients with metastatic melanoma [78] demonstrated a superiority of BRAF/MEK inhibitors within the first 12 months, later changing to a superiority of anti-PD-1 alone or in combination with anti-CTLA-4 with 3-year OS rates of 41% for BRAF/MEK inhibitors, 50% for anti-PD-1 alone, and 58% for anti-PD-1 plus anti-CTLA-4. This observation reflects the primary resistance under ICIs, the acquired resistance under BRAF/MEK inhibitors and the higher frequency of durable responses under ICI treatment.

According to the ESMO consensus conference [20], first-line decisions between BRAF/MEK inhibitors or ICIs should be individualized and based on performance status, organs involved, LDH level, tumor burden, tumor progression kinetics, comorbidities, patient preference, and treatment goals (short-term versus long-term benefit). For patients requiring urgent treatment, the rapid clinical response observed with BRAF/MEK inhibitors may provide rapid disease control. Furthermore, the immunological effects of BRAF/MEK inhibitors may offer a chance for an early switch to ICIs [79]. Patients who can be treated with ICIs for the first few months should be considered for ICIs first, providing the chance for long-term disease control even after treatment discontinuation. Overall, the optimal sequencing strategy is yet to be established.

### 6.3. Novel Sequencing Approaches for BRAF/MEK Inhibitors and Immune Checkpoint Inhibitors

To study sequencing approaches, prospectively planned clinical trials randomize patients in advance to pre-defined sequences of systemic therapies (e.g., SECOMBIT, NCT02631447 and EBIN, NCT03235245).

In the ongoing phase 3 DREAMseq trial, patients with unresectable stage III or IV BRAFV600-mutant melanoma receive either BRAF/MEK inhibitor therapy with dabrafenib and trametinib and switch after progression to ICI therapy with ipilimumab and nivolumab, or the other way around [80]. The completion of the trial is estimated in 2022.

The phase 2 ImmunoCobiVem trial enrolled patients with unresectable or metastatic BRAFV600 mutant melanoma to receive BRAF/MEK inhibitor therapy with vemurafenib and cobimetinib for three months [81]. All patients who did not show disease progression were randomized to continue with vemurafenib and cobimetinib until PD, followed by anti-PD-L1 therapy with atezolizumab (arm A), or to receive atezolizumab until PD, followed by vemurafenib and cobimetinib (arm B). Results of these trials are eagerly awaited.

### 6.4. Novel Combinations of BRAF/MEK Inhibitors and Immune Checkpoint Inhibitors

Given the rapid and deep responses seen with BRAF/MEK inhibitors and the durable responses observed with ICIs, the combination of these therapeutic strategies appears promising. The phase 3 IMspire 150 trial (NCT02908672) combined atezolizumab, an anti-PD-L1 monoclonal antibody, with the BRAF/MEK inhibitor combination vemurafenib and cobimetinib in patients with BRAFV600-mutant metastatic melanoma [42]. Further, 514 patients were randomly assigned to receive atezolizumab, vemurafenib and cobimetinib or placebo, vemurafenib and cobimetinib, as first-line therapy. The study met its primary endpoint of PFS. Although there was no difference in the PFS curves for the first 6–8 months, the curves separated thereafter. The triple therapy did not increase the ORR (Table 1). Grade 3 or 4 TRAEs occurred in 79% of patients treated with the triple combination and in 73% of patients treated with vemurafenib plus cobimetinib only. In conclusion, the addition of atezolizumab to targeted therapy with vemurafenib and cobimetinib was tolerable and significantly increased PFS in patients with BRAFV600-mutant metastatic melanoma.

A triple therapy of the novel anti-PD-1-directed monoclonal antibody spartalizumab in combination with dabrafenib and trametinib was studied in the phase 3 COMBI-I trial (NCT02967692), compared to dabrafenib and trametinib plus placebo, as first-line therapy in patients with BRAFV600E/K-mutant unresectable (stage IIIC) or metastatic (stage IV) melanoma (Table 1) [43]. With an ORR for the triplet therapy being slightly higher (69%) compared to the doublet (64%), the trial unexpectedly did not meet its primary endpoint of investigator-assessed PFS for patients treated with the triple therapy (Table 1), although median PFS for the triplet was 4.2 months longer than for the doublet. TRAEs were however considerably higher for the triplet: grade 3 or 4 TRAEs occurred in 55% of patients in the triple therapy arm compared to 33% in the dabrafenib/trametinib arm; TRAEs leading to discontinuation of all study drugs occurred in 12% compared to 8% of patients, respectively.

In order to reduce the increased risk of toxicity when continuously combining ICIs with BRAF/MEK inhibitors, the phase 2b trial IMPemBra investigated the clinical benefit of intermittent, short-term BRAF/MEK inhibition during anti-PD-1 therapy—based on preclinical findings that short-term, but not long-term, BRAF/MEK inhibition induced strong T cell infiltration, acting hence synergistically with anti-PD-1 monoclonal antibodies [82]. Thiry-two patients with BRAFV600-mutant metastatic melanoma received pembrolizumab and were randomized in week 6 either to continue pembrolizumab monotherapy (cohort 1) or to add intermittent dabrafenib and trametinib 2 × 1 week (cohort 2) or 2 × 2 weeks (cohort 3) or continuously for 6 weeks (cohort 4). ORRs at week 18 were 62%, 75%, 75%, and 50% in cohort 1, 2, 3, and 4, respectively. Grade 3 or 4 TRAEs were reported in 12%, 12%, 50%, and 62% of patients. The authors concluded that the combination of pembrolizumab plus intermittent BRAF/MEK blockade with dabrafenib and trametinib might be more feasible and tolerable than continuous triple therapy.

### 6.5. Combination of Targeted Therapy with ICIs in BRAF-Wildtype Melanoma

There is some pre-clinical evidence that in BRAF-wildtype melanoma, the combination of MEK inhibitors with ICIs may enhance anti-tumor effects. Based on these findings, IMspire 170, a phase 3 trial, randomized 446 patients with unresectable stage III/IV BRAF-wildtype melanoma to receive either the MEK inhibitor cobimetinib plus atezolizumab, or the anti-PD-1 monoclonal antibody pembrolizumab alone [83]. The trial did not meet its primary endpoint, showing a median PFS of 5.5 months with cobimetinib plus atezolizumab vs. 5.7 months with pembrolizumab alone. Regarding the safety profiles, grade 3 or 4 TRAEs and discontinuation of treatment occurred in 67% and 12% of patients receiving the combined therapy versus 33% and 6% receiving pembrolizumab monotherapy.

### 6.6. Summary

The first trials investigating combinations of BRAF/MEK inhibitors and ICIs yielded conflicting results in terms of PFS. Toxicity appears to be higher for the triplet therapies. For sequencing BRAF/MEK inhibitors and ICIs, reliable clinical data are still awaited.

## 7. Novel Drugs and Combinations

### 7.1. Introduction

Besides the already existing regimens, there is a variety of novel promising treatment strategies for patients with metastatic melanoma. Most of them *aim to elicit* an immune response against cancer cells. For example, NKTR-214 is a prodrug of IL-2 that stimulates expansion of CD8+ T cells, CD4+ T cells, and natural killer cells. IMCgp100 is a so-called ImmTAC (immune-mobilizing monoclonal T cell receptor against cancer) and acts as a link to redirect T cells to melanoma cells. In adoptive T cell transfer, a large number of antitumor lymphocytes is produced in vitro. After lymphodepletion, they are meant to serve as a highly personalized cancer therapy.

Combination of anti-PD-1 monoclonal antibodies with pegylated engineered interleukin-2 (IL-2).

In 1984, a 33-year-old woman, suffering from metastatic melanoma and progressing despite multiple prior therapies, was the first one receiving an infusion of aldesleukin (rIL-2), a recombinant version of IL-2. Only a few months later, the patient was free of disease. Being in CR over the years, this woman was the first to prove that it is possible to cure cancer by bolstering the patient’s immune system [84]. Later, aldesleukin was approved by the FDA as a treatment for metastatic melanoma based on pooled results of eight single-arm phase 2 trials. Interleukin had resulted—despite major toxicity concerns— in long-term survival in some patients, with “tail-of-the-Kaplan–Meier-curve” efficacy of a few percent, i.e., demonstrating long term recurrence-free survival. IL-2 leads to potent activation of immune cells, and in particular, to expansion of CD8+ T cells. Nevertheless, due to frequent and unchecked induction of the IL-2 cascade, this procedure is associated with serious AEs, e.g., hypotension, dyspnea, renal insufficiency, hepatic dysfunction, cardiac arrhythmias, and neurotoxicity [85,86].

Today, next-generation aldesleukin-derivates like bempegaldesleukin (BEMPEG, NKTR-214), a human recombinant prodrug of IL-2, are under clinical investigation. BEMPEG is based on the IL-2 core with 6 releasable polyethylene glycol (PEG) chains attached. With fully pegylated IL-2 hardly showing biological activity, the partial pegylation and slow release of PEG chains, as demonstrated in vivo, leads to the formation of IL-2 conjugates, selectively stimulating CD8+ T cells, CD4+ T cells, and natural killer cells without increasing regulatory T cells within the tumor microenvironment [87]. Studies indicate that BEMPEG also leads to overexpression of ICOS, PD-1, and CTLA-4, implying an activation of immune cells [88]. Strong synergistic effects were demonstrated in mouse tumor models for the combination of NKTR-214 and an anti-CTLA-4 monoclonal antibody [87].

In the phase 1 PIVOT-02 trial, 38 immunotherapy-naïve patients with solid tumors including melanoma received BEMPEG and nivolumab [89]. The combination achieved an ORR of 59.5%, with 18.9% CRs. Flu-like symptoms (86.8%), rash (78.9%), fatigue (73.7%), and pruritus (52.6%) were common adverse events with the combination; for 21.1% of patients, grade 3 or 4 TRAEs were reported. Changes in immune signatures characteristic for treatment response, e.g., an accumulation of interferon-γ or CD8+ TILs, were demonstrated by biomarker analysis of baseline tumor biopsies. Responses were also observed in patients with a tumor microenvironment deemed unfavorable for immunotherapy.

As PIVOT-02 yielded strong synergistic effects, Bristol-Myers Squibb launched a phase 3 trial comparing NKTR-214 and nivolumab with nivolumab monotherapy [90]. The primary completion is estimated in 2024 and results have not yet been published.

### 7.2. Exploration of Novel Immunotherapeutic Targets: gp100

**Figure 4:**
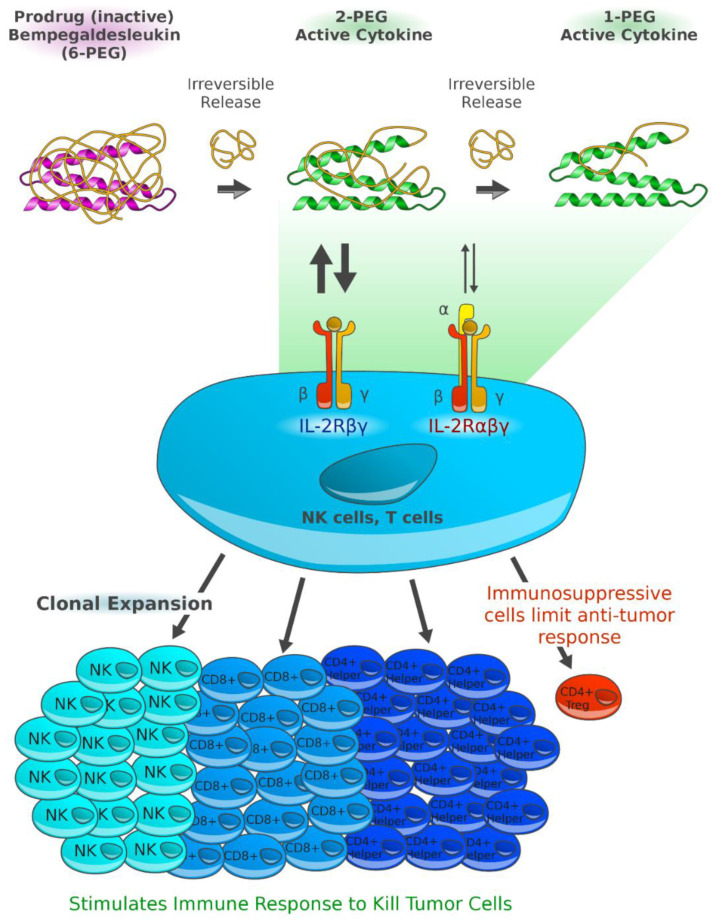
Proposed mechanisms of action of BEMPEG. After irreversible release of its six releasable polyethylene glycol (PEG) chains alternating its pharmacokinetic properties and its receptor binding, bempegaldesleukin is thought to expand and to activate CD8+ effector T cells and natural killer (NK) cells over T-regulatory cells (Tregs) [88]. The generation of active IL-2 conjugates with limited binding ability to the IL-2Rα subunit, thereby favoring the formation of dimeric βγ-IL-2 receptors (IL-2Rβγ; CD122), may explain its immunological activity investigated in vivo. Figure adapted from Charych D [94] and created by Gellrich FF, first published in *J. Clin. Med.* [23].

Glycoprotein (gp)100 (Figure 4) is a melanocyte-lineage specific antigen highly expressed in melanoma cells. Expressed gp100 in the context of HLA-A*0201 was seen to be detected and lysed by TILs [91]. However, the rate of recognition of self-derived antigens, as e.g., overexpressed in tumors, is low, due to a weak affinity of the T cell receptor (TCR) in order to reduce autoreactivity [92]. Immune-mobilizing monoclonal TCRs against cancer (ImmTACs) consist of a tumor-epitope-directed TCR and a CD3-specific, humanized single-chain antibody fragment and are thought to act as a link between tumor and T cells [93]. In melanoma, the domain of IMCgp100 (tebentafusp) exclusively binds to gp100 and might therefore redirect T cells to melanoma cells due to its CD3 antibody fragment.

Based on data of IMCgp100–202, a phase 2 trial, comparing tebentafusp with investigator’s choice (dacarbazine, ipilimumab, or pembrolizumab), the agent gained fast track designation for the treatment of metastatic uveal melanoma by the FDA [95]. In phase 1 of the IMCgp100–102 study, escalating doses of IMCgp100 were investigated in 19 heavily pre-treated patients with metastatic uveal melanoma, elevated LDH (87%) and liver metastases (100%) [96]. The 1-year OS rate was 74%. The IMCgp100-201 trial, a phase 1b/2 study, investigates tebentafusp alone or in combination with ICIs in patients with metastatic cutaneous melanoma [97]. The primary completion is estimated in 2022.

### 7.3. Adoptive T Cell Transfer

The idea of adoptive T cell transfer is to produce and select a large number of antitumor lymphocytes that recognize tumor cells with strong affinity. Moreover, inhibitory factors that are normally existing in vivo, are removed through in vitro activation [98]. One of the major limiting factors is the identification of cells selectively targeting tumor antigens and not the ones expressed on normal tissues [98].

The resected melanoma is split up into multiple tumor fragments individually grown in IL-2. Overgrowing lymphocytes lyse the tumor and pure cultures of lymphocytes with reactivity against the tumor arise. In around 5–6 weeks, it is possible to cultivate up to 10**^11^** lymphocytes. An additional lymphodepletion appears to be able to increase the T cell persistence and the clinical response including the DOR [99].

At the ASCO 2020, data of C-144-01, a phase 2 trial including 66 patients with unresectable metastatic melanoma who have progressed on ICIs and BRAF/MEK inhibitors, were shown [100]. Tumor tissue was resected and TILs were produced ex vivo. The patients underwent nonmyeloablative lymphodepletion with cyclophosphamide followed by fludarabine; then they received one-time treatment of expanded and activated TILs and up to 6 doses of IL-2. The ORR was 36%, with 3% CR and 33% PR. The disease control rate was 80%; after a median follow up of 18.7 months, the median DOR has *not been reached*. Toxicity, most likely due to lymphodepletion and IL-2, was high, with grade 3 or 4 TRAEs in 97% of patients. Thrombocytopenia, anemia, and neutropenia, lasting a few weeks, were the most common AEs. One death was caused by intraabdominal hemorrhage and was considered to be possibly related to TILs. The other death was due to acute respiratory failure and not related to TILs per investigator assessment.

### 7.4. Summary

Despite the tremendous clinical therapeutic advantage induced by ICIs and BRAF/MEK inhibitors, further improvements in the therapy of patients with advanced melanoma are needed. Apart from interdisciplinary attempts to improve the outcome of melanoma patients who were either initially diagnosed with prognostically highly unfavorable brain metastases—as referred to in the following outlook—or experienced brain metastases in the further course of their disease, the development of novel therapeutics remains a priority. Here, novel immunological approaches to improve further current melanoma therapy may play a central role, as highlighted by the examples described above. However, for the moment, these novel forms of therapy are just experimental and have to confirm their effectiveness in large, prospectively planned single-arm trials or, even better, in randomized controlled trials.

## 8. Outlook: Treatment of Melanoma Brain Metastases (MBM)

Melanoma often leads to the formation of brain metastases; if present, prognosis for melanoma patients is truly dismal with a median OS of about 4 months. Local therapy is a cornerstone of therapy for brain metastases including surgical resection, stereotactic radiosurgery (SRS) or hypo-fractionated stereotactic radiotherapy (hfSRT), or with decreasing relevance, whole brain radiotherapy (WBRT) [101].

For MBM patients, the use of ICIs or anti-BRAF-directed targeted therapy has remarkably improved outcomes with OS medians reported of about 7 months for anti-CTLA-4 ipilimumab [102], of around 10 months for anti-PD-1 pembrolizumab or nivolumab [103] and of up to 24 months for combined BRAF/MEK inhibition [104]. For combined immune checkpoint blockade by ipilimumab plus nivolumab, a 3-year OS rate of 49% in patients with asymptomatic brain metastases was recently reported. [103,105].

### 8.1. Dabrafenib Combined with Trametinib

The phase 2 COMBI-MB study enrolled 125 patients with BRAFV600-mutant melanoma and brain metastases in four cohorts receiving dabrafenib and trametinib: BRAFV600E-mutant, asymptomatic MBM without prior local brain therapy (cohort A; 76 pts) or with prior local brain therapy (cohort B; 16 pts), BRAFV600D/K/R-mutant, asymptomatic MBM without or with prior local brain therapy (cohort C; 16 pts) and BRAFV600D/E/K/R -mutant, symptomatic MBM without or with prior local brain therapy (cohort D, 17 pts) [106]. After a median follow-up of 8.5 months, intracranial response was achieved in 58% of patients in cohort A, 56% in cohort B, 44% in cohort C, and 59% in cohort D. The 6-months OS rates were 79%, 81%, 69%, and 88%, respectively. Intracranial RRs were nearly the same in patients with asymptomatic and symptomatic MBM. However, the median intracranial DOR was 6.5 months for asymptomatic patients and 4.5 months for symptomatic patients compared to the median DOR of 12–14 months for patients with metastatic melanoma without MBM. Explanations for the short DOR in the brain include incomplete MAPK-pathway inhibition, potentially due to poor drug penetration into the brain and/or microenvironmental factors in the brain [107,108]. The toxicity profile was in line with previous trials in patients with metastatic melanoma without MBM.

### 8.2. Nivolumab Combined with Ipilimumab

CheckMate-204 investigated the efficacy of nivolumab plus ipilimumab in a prospectively conducted trial; updated results were reported at the ASCO Annual Meeting 2019 [109]. The phase 2 trial enrolled MBM patients with asymptomatic brain metastases and without steroid treatment (cohort A) and MBM patients with symptomatic brain metastases with or without steroid treatment (cohort B). All patients received 4 cycles of each ICI, followed by nivolumab monotherapy until severe toxicity or tumor progression. The intracranial and extracranial responses of 101 patients with asymptomatic MBM were largely consistent with an intracranial ORR of 54% after a median follow-up of more than 20 months. The OS rate in this cohort A was 75% after 18 months, while the median OS was not yet reached. In cohort B, an intracranial ORR for 20 patients recruited with symptomatic MBM was 17% after a median follow-up of 5 months; a median OS of 8.7 months was reported here. In both cohorts, more than 50% of patients experienced grade 3–4 TRAEs. The rate was comparable to patients without brain metastases [48].

A second phase 2 trial conducted by the Melanoma Institute Australia enrolled 76 patients with MBM in three cohorts. The ABC trial recruited for cohorts A and B patients with asymptomatic MBM with no prior local brain therapy who were administered either nivolumab plus ipilimumab, or nivolumab.

In cohort C, previously treated patients, patients with neurological symptoms or patients with leptomeningeal disease (LMD) were treated with nivolumab. Intracranial ORR was 51% (cohort A), 20% (cohort B), and 6% (cohort C). The 3-year OS rates were 49% (cohort A), 42% (cohort B), and 19% (cohort C) [103,105]. Grade 3 or 4 TRAEs occurred in 54% of patients, respectively in 20% of patients (cohort B) and 13% of patients (cohort C).

### 8.3. Combination of Systemic Therapy and Radiosurgery

BRAF/MEK inhibitors and anti-CTLA-4/anti-PD-1 antibodies demonstrate high RRs and have significantly improved the prognosis of patients with MBM. However, the majority of patients with MBM, in particular patients with symptomatic MBM, still progress and die. Multiple retrospective studies suggest that the combination of systemic therapy with radiotherapy represents an approach to further improve the outcome for MBM patients [110].

In a German retrospective trial [111], for example, data of 380 patients with MBM treated with nivolumab plus ipilimumab were analyzed. Further, 31% had symptomatic MBM. The median follow-up was 18 months. Local therapy with SRS or surgery led to a significant improvement in OS compared with not receiving local therapy. Patients who received local therapy reached a median OS of 24 months compared with 16 months in patients without local therapy. Notably, only 4.5% of the local therapy group received only surgery. Altogether, immunotherapy with nivolumab plus ipilimumab, particularly in combination with SRS improves OS in both asymptomatic and symptomatic MBM.

In a French multicenter prospective study [104], clinical data were collected from 262 patients with MBM. Remarkably, 41% of patients demonstrated neurological symptoms and 41% were treated with corticosteroids. In addition, 35% of patients received combined radiotherapy (cRT) and 65% did not. Further, 69% of patients treated with cRT and 60% of patients without cRT were treated with immunotherapy and 31% and 40% with targeted therapy, respectively. After a median follow-up of 6.9 months, the median OS in the cRT group was significantly longer with 16.8 months than in the no-cRT group with 6.9 months. No increasing rate of toxicities was observed in the RT group.

Ongoing prospective studies investigate combinations of SRS with BRAF/MEK inhibitors or ICIs. Notably, an ongoing phase 2 randomized trial investigates ipilimumab plus nivolumab with concurrent intracranial SRS versus ipilimumab plus nivolumab alone in patients with asymptomatic MBM (NCT03340129).

### 8.4. Combination of Targeted Therapy with Immune Checkpoint Inhibitors

Prospective trials currently investigate combined regimens with BRAF/MEK inhibitors plus ICIs in MBM patients, such as TRICOTEL (NCT03625141) or TRIDeNT (NCT02910700). The rationale of the triple strategy is to combine the rapid response and high RRs of BRAF/MEK inhibitors in both asymptomatic and symptomatic MBM with the durable response of ICIs.

### 8.5. Intrathecal Immunotherapy

Metastatic melanoma patients with LMD have a poor prognosis with a median OS of less than 3 months.

Recently, first results for the intrathecal application of nivolumab in LMD were reported. In this phase 1 study, increasing doses of 5, 10, and 20 mg nivolumab were injected intrathecally, in addition, nivolumab was given intravenously. To date, in 18 patients, this treatment was feasible and well tolerated. Median OS was approximately 5 months. Recruitment in this trial is ongoing with increasing dose levels [112].

### 8.6. Summary

MBM are historically associated with poor prognosis. In patients with asymptomatic MBM, the anti-PD-1 monoclonal antibody nivolumab in combination with the anti-CTLA-4 monoclonal antibody ipilimumab achieved a high rate of sustained intracranial responses, supporting nivolumab plus ipilimumab as first-line therapy in these patients. Treating patients with symptomatic MBM remains challenging. In the presence of a BRAFV600-mutation, high RRs were demonstrated with the BRAF/MEK inhibitor combination dabrafenib plus trametinib. However, the intracranial DOR is limited. Some patients may benefit from nivolumab plus ipilimumab. Retrospective studies suggest that the combination of SRS with BRAF/MEK inhibitors and in particular with ICIs extends OS without increasing toxicity. The combination of SRS with BRAF/MEK inhibitors but also ICIs, and of BRAF/MEK inhibitors in line with ICIs, is currently investigated in ongoing, prospective clinical trials.

## 9. Conclusions

Clinically significant advances in the systemic therapy of metastatic melanoma have been achieved over the past decade. Combined ICIs (nivolumab plus ipilimumab) and combined BRAF/MEK inhibitors (dabrafenib/trametinib, encorafenib/binimetinib, vemurafenib/cobimetinib) were the first combinations to show superior OS compared to monotherapy. Patients treated with nivolumab combined with ipilimumab achieve a 5-year survival rate of more than 50% but at a cost of high toxicity with very rare cases of fatal outcome.

Treatment outcomes for patients with metastatic melanoma have greatly improved over the last decade, however, treatment responses in many patients continue to be heterogeneous and of short duration. Further preclinical and clinical research is required. Several novel therapeutic strategies are currently being explored. These include new immunotherapy combinations such as ICIs with other immunotherapies (e.g., pegylated interleukin-2, talimogene laherparepvec) and targeted therapy combined or sequenced with immunotherapy (e.g., triplet combination of anti-BRAF/MEK targeted therapy with ICI). Ongoing clinical trials and novel development approaches focus on poor prognosis groups (e.g., anti-PD-1 refractory melanoma, brain metastases, and uveal melanoma) in order to further ameliorate outcomes of systemic therapy and to further improve long-term survival for patients with metastatic melanoma, even for those with a dismal prognosis today.
